# The influence of supervisor supportive behaviors on subordinate job satisfaction: the moderating effect of gender similarity

**DOI:** 10.3389/fpsyg.2023.1233212

**Published:** 2023-12-29

**Authors:** Antonia Mercedes García-Cabrera, Sonia María Suárez-Ortega, Francisco Javier Gutiérrez-Pérez, María José Miranda-Martel

**Affiliations:** Faculty of Economics, Business and Tourism, University of Las Palmas de Gran Canaria (ULPGC), Las Palmas de Gran Canaria, Spain

**Keywords:** job satisfaction, supportive behaviors, supervisor gender, gender similarity, European countries

## Abstract

This paper analyses the influence that different supervisor supportive behaviors have on subordinate job satisfaction, while considering the gender of individuals involved (supervisor and subordinate). The empirical evidence provided by a sample of 29,833 subordinates from 35 European countries collected by Eurofound through the European Working Condition Survey allows us to assert the following. First, subordinate job satisfaction depends on their perceptions about the supportive behaviors provided by their supervisors in terms of respect, giving recognition for a job well done, coordinating work, helping get the job done, and encouraging the professional development of the subordinate. Second, job satisfaction is affected by supervisor gender, although in the opposite direction as hypothesized, such that when the supervisor is a woman, subordinates report lower job satisfaction. Third, also contrary to our expectations, supervisor-subordinate gender similarity reduces, rather than increases, subordinate job satisfaction. Fourth, gender similarity, as expected, weakens the impact of several supervisor supportive behaviors on this job satisfaction (i.e., giving recognition, coordinating work, helping get the job done, and encouraging development). In terms of practical implications, this work suggests that it is advisable for supervisors to show supportive behaviors toward subordinates. In addition, because demonstrating respect at work is not moderated by gender similarity and seems to be the most impactful supportive behavior for enhancing job satisfaction, supervisors should pay particular attention to the respect of subordinates.

## 1 Introduction

The literature broadly highlights the relevance of the supervisor and their responsibility within organizations (Stewart and Wiener, 2021). Due to their liaison role between the organization and front-line employees, supervisors are responsible for establishing the daily working conditions of their subordinates (Wesolowski and Mossholder, 1997). They, for example, can provide subordinates with trust and respect, emotional support and assistance in job performance, fair allocation of resources and responsibilities, and constructive evaluation (Dale and Fox, 2008; Zhang et al., 2023). Because of this daily relational proximity between the two, the supervisor’s behaviors, particularly those deployed to support subordinates, will affect the attitudes of the latter toward work (Campione, 2014). In this respect, *social exchange theory* (Homans, 1958; Blau, 1964) suggests that supervisors can offer support to employees in exchange for greater commitment, motivation, and performance (LePine et al., 2002; Rockstuhl et al., 2012; Gaudet and Tremblay, 2017). However, previous studies have examined the broad concept of supervisor support as a single construct (Ariza-Montes et al., 2019; Artz et al., 2020; Kizuki and Fujiwara, 2020; Hauff et al., 2022) this approach could obscure the different influences of each supervisor supportive behavior on subordinates’ attitudes toward work, including the widely studied relationship between supervisor support and subordinate job satisfaction.

Certainly, previous literature highlights how such supervisor support improves subordinate job satisfaction (Qureshi and Hamid, 2017; Zhang et al., 2023). Satisfied employees are relevant to the company given the impact of satisfaction on employee behavior, productivity, and intention to leave the company (LePine et al., 2002; Campione, 2014; Dappa et al., 2019). For example, absence of job satisfaction is associated with a lower likelihood that employees will contribute to the achievement of organizational goals, as well as a greater likelihood that they will develop behaviors that are counter-productive for the company (Spector, 1997; Șulea et al., 2010).

Due to the consensus in the literature on the relevance of the supervisor to subordinate job satisfaction, it is of interest to understand how their *gender* can affect the relationship between supervisors’ supportive behaviors and subordinates’ attitudes towards work. Such understanding could be crucial in the current context of gradual incorporation of women into supervisory positions in companies. Specifically, the progressive advancement of women toward intermediate positions in the organizational hierarchy, both in Europe and United States, is worth mentioning. By consulting successive editions of the European Working Conditions Surveys conducted by the European Foundation for the Improvement of Living and Working Conditions (Eurofound), we can see how women, who held only 22.3% of the supervisory positions in Europe in 1995, have slowly increased their share in this hierarchical level (27.6% in 2001; 30.4% in 2005; 30.7% in 2010; 33.9% in 2015), reaching 35.1% by 2021. In the United States, since Gallup-a global analytic and advisory firm serving leaders and organizations - began measuring Americans’ preferences regarding the gender of their boss over the years, employees preferred their boss to be a man. We had to wait until 2017 to stop finding this preference. In 2017, most respondents said that gender made no difference to them; although 23% said they would prefer a male boss, this represented 10% points less than in 2014 and 43 points less than in the initial reading in 1953 (Brenan, 2017). Thus, although the overall evolution of the figures points to a reduction in the *gender gap* in supervisory positions in companies, equality is still far from being achieved (Eagly and Wood, 2013; Al Fardan and Maroch, 2021).

In this regard, while there is a relevant body of literature studying the role of the supervisor, we have found a reduced number of papers analyzing the possible impact of gender on the supervisor-subordinate relationship (Grissom et al., 2012; Campione, 2014), and most of them have limitations in terms of their reliance on small sample sizes and the inclusion of few countries. For example, some of this evidence comes from samples collected in specific professional contexts that must be considered in the interpretation of results (e.g., public education, nursing, the army, etc.) and/or at times in the past when certain stereotypical beliefs regarding gender roles were more marked than they are today-e.g., sample of 2003–2005 in Grissom et al. (2012); sample of 2005 in Schieman and McMullen (2008) or of 2007 in Campione (2014). In addition, these papers provide mixed evidence, since they do not allow us to conclude whether supervisor gender affects job satisfaction and the direction of that possible influence.

Moreover, previous literature has resorted to the *social role theory* (Eagly et al., 1995; Eagly and Wood, 2013) and the *similarity attraction framework* (Schieman and McMullen, 2008) to explain the role of supervisor gender - in the context of social stereotypes and prejudices concerning male and female conditions - in terms of, for example, interactions with subordinates (Stewart and Wiener, 2021). In this vein, some previous works have also explored the moderating role of supervisor-subordinate gender similarity in the study of supervisors’ impact on subordinates’ behaviors at work (Parent-Rocheleau et al., 2023).

Based on those previous works, it is reasonable to suppose that supervisor gender, as well as supervisor-subordinate gender similarity, could shape the supervisor-subordinate relationship concerning supportive behaviors and affect subordinates’ job satisfaction. However, there is a lack of previous research on these relationships that shed light on this relevant issue. For example, the impact of supervisor gender on subordinate job satisfaction is rarely introduced into the study models (Campione, 2014), and the closest study found is the recent paper by Li et al. (2023), which examines ambidextrous leadership and its impact on employee voice while considering the moderating effect leader-subordinate gender similarity.

In order to fill these research gaps, this paper examines the impact of supervisor supportive behaviors on subordinate job satisfaction, as well as whether such relationships could be affected by the supervisor’s and subordinate’s gender. More specifically, this study explores the following three research questions: (1) Do different supervisor supportive behaviors influence subordinate job satisfaction? (2) Does supervisor’s gender affect subordinate job satisfaction? (3) Does supervisor-subordinate gender similarity affect subordinate job satisfaction? (4) Does supervisor-subordinate gender similarity moderate the impact of supervisor supportive behaviors on subordinate job satisfaction?

## 2 Theoretical foundations of the study

### 2.1 Supervisor supportive behaviors as antecedents of subordinate job satisfaction

As an attitude, *job satisfaction* is widely understood as “a positive (or negative) evaluative judgment one makes about one’s job or job situation” (Weiss, 2002, p. 175). From a comparative perspective, job satisfaction can be understood as the set of *emotional reactions* that occur in employees when considering what they want to obtain from the company and what they actually get (Cranny et al., 1992). Thus, this satisfaction can be associated with the organization’s capacity to attract an employee (Dappa et al., 2019). In this regard, Wyrwa and Kaźmierczyk (2020) identified several extrinsic factors that the company can act on to stimulate job satisfaction (in particular, fair and equitable wages, adequate working conditions, positive relationships with superiors and co-workers, and appropriate leadership style). They indeed found numerous models recently developed “in which a set of distinguished factors, acting synchronously, triggers or increases the feeling of job satisfaction” (Wyrwa and Kaźmierczyk, 2020, p. 139).

However, there is still no academic consensus on the relative importance of such factors. For example, Lee and Park (2021), in their analysis of the successive waves of the Korean Working Conditions Survey, found that the factors with the greatest impact on employee working conditions changed over time, highlighting: the physical environment in 2006, adverse social behavior in 2010, occupational status in 2011 and 2014, and management quality in 2017. In this sense, studies that focus in particular on the supervisor’s influence are proliferating, not necessarily considering their gender, but showing that this person plays an essential role in job satisfaction (Stewart and Wiener, 2021; Zhang et al., 2023) and that supervisor supportive behaviors can generate and maintain job satisfaction among employees (Gilbreath and Benson, 2004; Qureshi and Hamid, 2017; Kizuki and Fujiwara, 2020; Hauff et al., 2022). Particularly, such supervisor support can help employees absorb the tension and stress felt while performing their roles (House, 1981). Accordingly, supervisor support can be considered an important job resource that help employees to deal with high job demands (Bakker et al., 2007; Bakker and Demerouti, 2007) and this could eventually affect subordinate job satisfaction.

Consequently, this paper first focuses on the effect that different supervisor supportive behaviors can have on job satisfaction. To this end, and following Fritz and van Knippenberg (2020), we consider the supervisor in the broad sense, as the direct superior or immediate boss in the organizational hierarchy to whom the employee reports.

Supervisor supportive behaviors contribute to a climate of trust and respect at work, enhance emotional support, and help in job performance (Dale and Fox, 2008). Therefore, such behaviors are part of the *social support* that employees can receive from their company (Paustian-Underdahl et al., 2013; Ganesh and Ganesh, 2014). In particular, Paustian-Underdahl et al. (2013) highlight how such support can be defined as the perceptions of subordinates concerning the extent to which supervisors value their contributions and care about their personal and professional needs. In line with this definition, public surveys of working conditions (e.g., European Working Condition Survey [EWCS]) have included at least six supportive behaviors of the immediate boss which have, in turn, been considered in various studies, albeit as an aggregate construct (Ariza-Montes et al., 2019; Artz et al., 2020; Kizuki and Fujiwara, 2020; Hauff et al., 2022): (i) respects you as a person (hereafter, “respect”), (ii) gives you praise and recognition when you do a good job (hereafter, “giving recognition”), (iii) is successful in getting people to work together (hereafter, “coordinating work”), (iv) is helpful in getting the job done (hereafter, “helping get the job done”), (v) provides useful feedback on your work (hereafter, “providing useful feedback”), and (vi) encourages and supports your development (hereafter, “encouraging development”).

The interactions between supervisors and subordinates concerning such supportive behaviors lead us to understand the relationship between these two groups as a *social exchange*, which is discretionary in nature and based on mutual trust, where the supervisor voluntarily offers their support to employees in exchange for a greater commitment, motivation and/or performance on their part (Gaudet and Tremblay, 2017). The *social exchange theory* (Homans, 1958; Blau, 1964) proposes that the supervisor’s discretionary behavior will be better valued by employees than any other resource offered by the organization by obligation. However, and considering the *job demands-resources model*, some of those supportive behaviors could be also considered by the subordinate as a job demand. Although job demands are not necessarily negative, they may become a “job stressor” when meeting such demands is challenging for the employee (Bakker et al., 2007; Bakker and Demerouti, 2007). For instance, the supervisor’s feedback could be perceived by the subordinates as a call for improving the way they are doing the task, and could be a stressor. In this respect, London and Smither (2002) found that an organizational culture which is supportive of feedback processes could be necessary for such feedback to lead to improve satisfaction. Accordingly, it is pertinent to consider the impact of each supervisor supportive behavior separately.

For example, and concerning respect, working under the supervision of a leader who treats subordinates with respect is one of the most valued factors for subordinates in their daily work (Decker and van Quaquebeke, 2015). Certainly, when employees were queried about the qualities they value most in their jobs, they prioritized having a supervisor who treats them respectfully over other factors such as autonomy, job security, and a high income (van Quaquebeke et al., 2009). Coherently with such relevant findings, while responding to the socio-emotional need for respect, supervisors may encourage employees in their charge to consolidate a sense of belonging and social identification with the organization (Gaudet and Tremblay, 2017; Dappa et al., 2019).

In addition, employees who are recognized for their work by their supervisors feel supported by the organization and will have greater confidence in their valued contributions to their firms (Abdullah et al., 2016) and that they will continue to be recognized in the future (Ganesh and Ganesh, 2014; Artz et al., 2020; Pfister et al., 2020). Besides, according to Pfister et al. (2020), the frequent positive affective experiences associated with recognition at work and the belief that recognition is a rather stable feature of an individual’s work situation should contribute to the overall positive evaluation of an employee’s job that constitutes job satisfaction. Coherently, when employees receive recognition for their work, such recognition can operate as a source of intrinsic motivation at work that give rise to increased job satisfaction (Abdullah et al., 2016).

Moreover, the supervisor, as the person responsible for coordinating the work, must also lead the proper integration of the team members, establish inspiring goals, and provide the necessary resources to organize the team - aspects that can also strengthen subordinate job satisfaction (Dappa et al., 2019). Supervisors can also provide employees with technical assistance so that they can demonstrate and transfer their skills, knowledge, and positivity to the organization (Dale and Fox, 2008; Qureshi and Hamid, 2017). Likewise, constant and fluid communication is key to this “win-win” social exchange relationship between supervisor and employee, especially regarding the supervisor’s useful and effective feedback on the results achieved (Paustian-Underdahl et al., 2013; Yuan et al., 2018). Finally, another possible example of a supervisor’s supportive behavior would be the deployment of their skills to motivate and guide employees in their need for professional development, so that they can achieve their personal goals and, at the same time, contribute to the achievement of the organization’s strategic objectives (Gilbert et al., 2011; Hauff et al., 2022).

In view of the above, a first research hypothesis is proposed:

*H1*: The greater the supervisor’s supportive behavior in terms of respect (*H1*a), giving recognition (*H1*b), coordinating work (*H1*c), helping get the job done (*H1*d), providing useful feedback (*H1*e), and encouraging development (*H1*f), the higher the subordinate’s job satisfaction.

### 2.2 Gender, supervisor supportive behaviors, and subordinate job satisfaction: direct and moderating effects

Social role theory attributes stereotypical behaviors to individuals according to their gender (Eagly and Wood, 2013). These stereotypes, since they are based on biological attributes and rooted in society, operate as culturally shared beliefs that establish expectations about how women and men are and how they should behave, so such stereotypes “[…] can be both descriptive and prescriptive in nature” (Gipson et al., 2017, p. 35). The premise of this theory is that those stereotypes also exist for the different roles an individual can exert. Considering the leading role, according to Eagly et al. (1995) male leaders are perceived to be better than female leaders when exercising roles that are more consistent with the male gender role (i.e., ability to direct and control people), while women are better for roles that are more consistent with the female gender role (i.e., ability to cooperate and maintain good relationships with others). In addition, it appears that men and women respond differently to various aspects of social relations, showing “agentic traits”, i.e., is a hard worker, assertive, independent, self-sufficient, individualist, ambitious, dominant, competitive, etc., or “communal qualities,” i.e., is selfless, caring, sociable, interdependent, considerate, connected, family oriented, etc., (Bakan, 1966) - women tend to obtain a higher score for *communal traits* than men (Eagly, 2009). Specifically, female leaders show empathy and build relationships more easily than men (Fletcher et al., 2000). In addition, Eagly and Karau (2002), and based on the *role congruence theory* and the *stereotypic fit hypothesis*, assert that group members (e.g., men, women) could experience discrimination in different social roles or positions when their group stereotypically does not have characteristics associated with success in those positions.

From this perspective, supervisor gender, as a visible feature which subordinates can perceive, may become a key variable that influences their perception of the immediate superior (Li et al., 2021). In this regard, Campione (2014) points out that subordinates sometimes notice the visible characteristics of supervisors, including gender, as a means to infer the expected actions of superiors (e.g., in cases where there is insufficient work experience, where there is pressure to make a rapid assessment of the superior), and, based on such inferences, they make assessments of the working conditions that affect them, which trigger a certain level of job satisfaction. Indeed, these descriptive and prescriptive stereotypes, very present in social interactions, can lead to biased judgements and, therefore, alter the perception regarding male and female supervisors (Gipson et al., 2017).

The paper of Hilton et al. (1995) on supervisor race as a visible and observable trait acquires interest in this argument since the authors find that, even in cases where the supervisor deploys supportive behaviors toward the subordinate, this may not improve the job satisfaction of the latter. This idea is supported by some papers which have found that, regardless of the managerial style deployed by the supervisor, the supervisor’s gender will influence the attitudes and job satisfaction of the employees in the company (Grissom et al., 2012). Thus, as Campione (2014) states, by analyzing whether the supervisor’s gender directly influences subordinate job satisfaction, we are identifying the subordinate’s possible preference towards a particular gender - a preference based on social expectations that reflect stereotypes and prejudices and have an impact on the workplace.

There are, however, differing views on the direction of this influence. While some studies seem to predict greater job satisfaction when the supervisor is a man (Schieman and McMullen, 2008; Grissom et al., 2012), others identify greater satisfaction when the supervisor is a woman (Grissom et al., 2012), and others find no impact of the supervisor’s gender on the subordinate’s job satisfaction (Campione, 2014). Among the factors to which these mixed evidences are attributed, we can mention the professional context of study, e.g., nursing, education, army (Eagly and Carli, 2003), the representation of one gender in relation to the other in the company (Wesolowski and Mossholder, 1997; Peccei and Lee, 2005; Steffens et al., 2019), and the supervisor-subordinate gender (dis)similarity (Campione, 2014). Since this current article is interested in the influence of gender on subordinate job satisfaction, we first focus on the supervisor’s gender as a visible characteristic, and subsequently on the gender similarity between the supervisor and the subordinate.

Concerning the direct effect of the supervisor’s gender, Blau (1994) states that the relevance of this visible trait is such that it may obscure other structural effects (e.g., occupation, industry, dominance of one gender in the organization) on job satisfaction. Taking up the precepts of the *social role theory* (Eagly et al., 1995; Eagly and Wood, 2013), subordinates can be expected to consider their superiors as having a certain personality and behaving in a particular way, according to existing gender stereotypes. In particular, since the literature notes that subordinates’ job satisfaction is affected by the social support they receive from their supervisor (Qureshi and Hamid, 2017), as well as the orientation of the latter toward relationships (Zenger and Folkman, 2012), existing stereotypes regarding behavior that men and women can display in this direction, regardless of actual behaviors, could impact subordinate job satisfaction (Stewart and Wiener, 2021), because the employees’ mere expectations of the support they would receive from their supervisor may shape their satisfaction (Penning de Vries and Knies, 2023). In this regard, compared with male leaders, women are considered to have greater empathy and capacity to build relationships (Fletcher et al., 2000), and more ability to cooperate and be more community oriented, e.g., supportive, sociable, considerate, family oriented (Bakan, 1966; Eagly, 2009), among other traits and behaviors. Table 1 offers additional arguments that reinforce the idea that women are more communal than men in their role as leaders. Based on this perspective, social role theory would indicate that by the mere fact that the supervisor was a woman, subordinates will attribute to her a way of being and acting that would have a positive impact on their job satisfaction, which leads us to propose the following:

**Table 1:** Gender differences concerning communal behaviors in the leadership literature.

Communal behavior	Gender differences	Author
Related to respect	Women, to a greater extent than men, make use of a soft tone in order not to look powerful or offensive.	Dappa et al. (2019)
Related to giving recognition	Female leaders generally grant promotions to their subordinates to motivate them.	Laub (1999)
Related to coordinating the work	Female leaders know how to build successful cooperative connections at work and create communities, and they are more likely than men to successfully attend to the coordination challenges presented in the organization.	Zenger and Folkman (2012) and Post (2015)
Related to helping get the job done	Women are better in the role of mentors, training their employees to help them eliminate self-limitations.	Laub (1999)
Related to providing useful feedback	Female managers, to a greater extent than men, monitor and provide feedback to employees, thus encouraging their efforts.	Teven (2007)
Related to encouraging subordinate’s development	Female leaders are better than men in developing others. Women inspire and motivate others better than men. Women, to a greater extent than men, encourage their subordinates to develop their full potential.	Cavallo and Brienza (2006), Zenger and Folkman (2012), Dappa et al. (2019)

*H2*: Supervisor gender will affect subordinate job satisfaction so that it will be higher when the supervisor is a woman than when it is a man.

As already highlighted, the literature also indicates that how the supervisor’s gender affects the subordinate’s job satisfaction could be influenced by the subordinate’s gender. This means that supervisor-subordinate gender (dis)similarity could also be influencing job satisfaction. In relation to this, some previous studies have pointed out that employees prefer to work with a supervisor of the same gender (Campione, 2014). In this vein, several theories offer arguments to justify why *gender similarity* could have a positive effect on subordinate job satisfaction.

First, the similarity attraction framework discussed by Schieman and McMullen (2008) proposes that “gender similarity” increases attraction and convergence of attitudes and priorities between supervisors and subordinates, and also fosters cohesion, as well as it reduces potential conflicts at work. Gender dissimilarity, on the contrary, would involve differences linked to deeply-rooted social stereotypes and prejudices. In the case of a “gender dissimilarity” between the supervisor and the subordinate, they may have to deal with negative prejudices about each other (Pelled et al., 1999), which would threaten cohesion and increase conflict at work (Tsui et al., 1992), so harming subordinate job satisfaction.

Second, taking up the precepts of the *social role theory* (Eagly et al., 1995; Eagly and Wood, 2013), we can also expect that subordinate job satisfaction will diminish in cases where the boss has the opposite gender. According to that theory, it can be expected that male and female employees will differ in the support they expect and wish to receive from their supervisors. This theory predicts that women are more likely to expect and value a strong interpersonal relationship with their supervisor, which helps to embed them into their job and promote positive work attitudes, as they are more people oriented (Johnson et al., 2008; Bellou, 2011). However, men’s lesser desire for communal activities-i.e., they are more task-oriented (Johnson et al., 2008; Bellou, 2011) - suggests that the interpersonal aspect of the *leader-member exchange* will be less salient for male subordinates (Collins et al., 2014). Accordingly, male subordinates may have their job satisfaction enhanced if they have a male supervisor, as they share *task-oriented* styles and independency within the supervisory relationship (Fritz and van Knippenberg, 2020), whereas female subordinates would feel their job satisfaction improved if they had female supervisors, as they share the *people-oriented* style. All of the above leads us to propose the following:

*H3*: Supervisor-subordinate gender similarity will affect job satisfaction so that subordinate job satisfaction will be higher when gender similarity exists.

According to the *social role theory*, it can be expected that male and female subordinates will differ in the level of support they expect and wish to receive from their supervisors, but also male and female supervisors will differ on the level of support they wish to deploy. Thus, supervisor-subordinate gender similarity emerges as a relevant factor that give rise to potential gaps and misunderstanding in the level of desirable support from the supervisor. These misunderstandings could harm the positive impact of supportive behaviors on job satisfaction.

In this vein, Graham et al. (2018) warn that supervisor-subordinate gender similarity is likely to lead to expectation bias between them, because both will assume the other to have attitudes and beliefs similar to their own, and that is not always so. When discrepancies between expectations exist, subordinates could feel frustration and dissatisfaction (Phillips and Loyd, 2006) due to falling short on expectations (Penning de Vries and Knies, 2023), as the expectation disconfirmation theory predicts (Ilgen, 1971). Therefore, expectations on the kind and level of supportive behaviors deployed by women and men, based on social stereotypes concerning gender roles, could harm the supervisor-subordinate relationship and, accordingly, subordinate job satisfaction.

Under this perspective, gender similarity could moderate the impact of supervisor supportive behaviors on subordinate job satisfaction. In particular, gender similarity could give rise to misunderstandings and conflicts due to unmet expectations (Li et al., 2021, 2023) that will weaken the positive relationship of supervisor supportive behaviors on subordinate job satisfaction. Accordingly, we posit that:

*H4*: Supervisor-subordinate gender similarity moderates the impact of supervisor supportive behaviors on job satisfaction, such that supervisor supportive behaviors will weaken the positive impact on subordinate job satisfaction when gender similarity exists.

**Figure 1:**
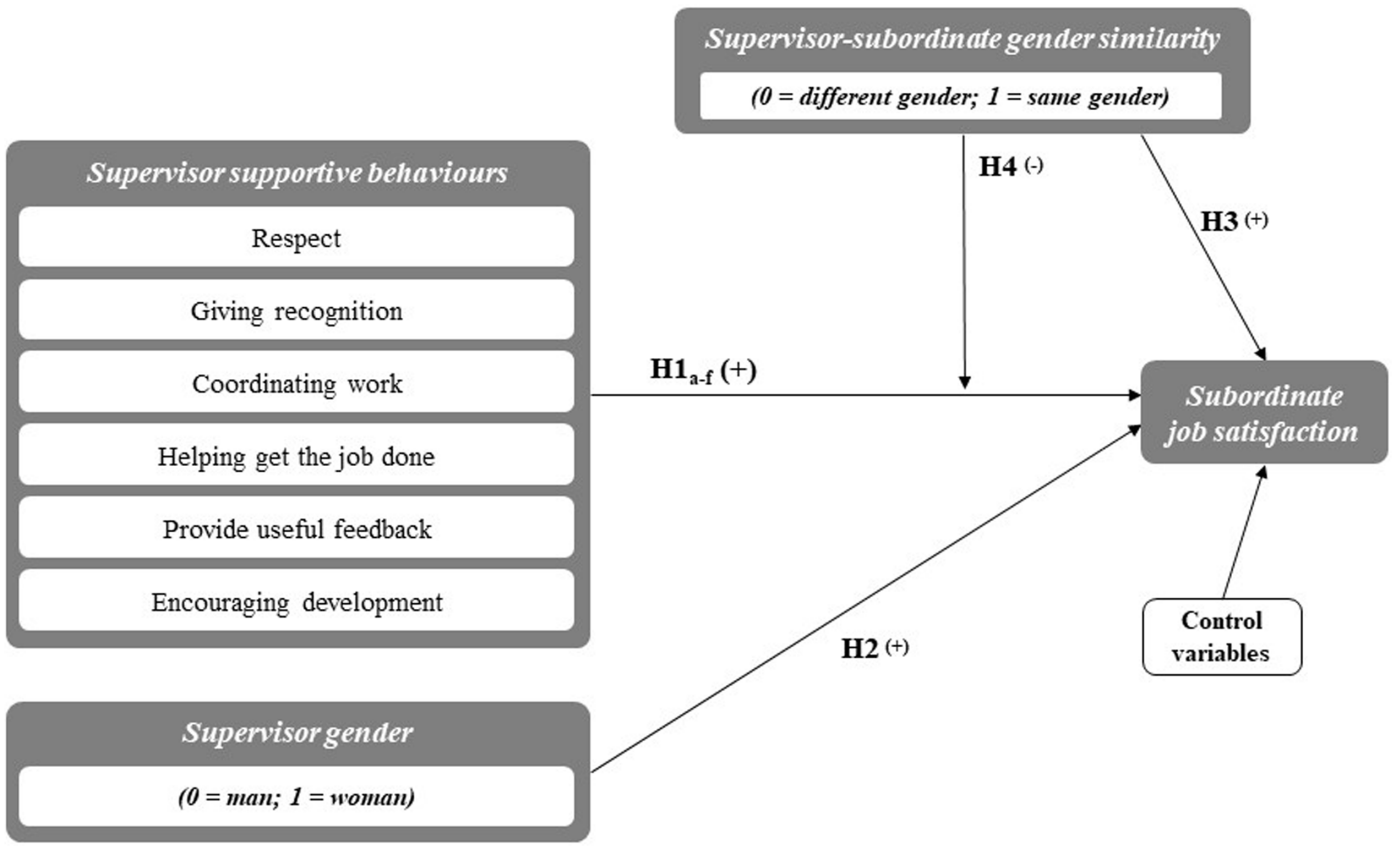
Graphical overview of the hypothesized effects.

Figure 1 provides a graphical overview of the hypothesized direct and moderating effects that are examined in the current study by following the method reported below.

## 3 Methodology

### 3.1 Data source and sample

The study sample was obtained from the sixth European Working Conditions Survey (EWCS) released by Eurofound in 2015, which is the most recent edition of this survey that considers each supportive behavior separately. The EWCS is a survey conducted among a statistical sample of workers who were interviewed face-to-face, comprising a cross-section of society in each country. Specifically, its sampling procedure employs a stratified multistage approach, ensuring a representative sample of the European workforce. This method guarantees that the survey covers all sectors and occupational groups. In each country, geographic regions and three levels of urbanization are used as sample criteria. In each stratum, a random sample of households is drawn, and in each household the selected respondent was the person who is employed and whose birthday is next. Sample validity is guaranteed using different types of weights to ensure the representativeness of the sample. Finally, and concerning quality of the survey, an external survey quality assessment reported that the quality of the sixth EWCS is very high, compared to similar surveys, and complies with international quality standards (see EWCS 2015-Methodology in https://www.eurofound.europa.eu/en/home for further information).

From the complete database that collects information provided by 43,850 workers from 35 European countries, this study has extracted a subset of data on workers who, not being supervisors of others, have a direct superior or immediate boss. Thus, the study sample covers a total of 29,833 subordinates representing the 35 countries, with frequency varying between 7.6% (Spain) and 1.7% (Albania).

Regarding the gender of the respondents, it is worth pointing out that 46.3% are male and 53.7% are female. The average age of the respondents is 41.8 years old (SD = 12.3). The level of education is varied: Early childhood education (0.4%), primary education (3.4%), Lower secondary education (13.1%), upper secondary education (44.3%), post-secondary non-tertiary education (7.2%), short-cycle tertiary education (9.4%), bachelor or equivalent (12.9%), master or equivalent (8.5%), and doctorate or equivalent (0.7%). The average seniority of the respondents in the current company is 9.17 years. Concerning *occupation*, technicians and professionals (29.8%) and service and sales (20.2%) account for half of the sample. Most of the respondents work for firms in the private sector (66.0%). Regarding *industry*, the sample represents the 21 sections of the NACE classification (rev. 1.1), the most frequent being the following: Wholesale and retail trade - including repair of motor vehicles and motorcycles - (14.9%), manufacturing (14.7%), human health and social work (11.5%), and Education (9.7%). In relation to *supervisor-subordinate gender similarity*, the sample analyzed comprises 11,802 cases of male–male (39.6%), 8,523 cases of female–female (28.6%), 7,503 cases of male supervisor-female subordinate (25.1%), and 2,005 cases of female supervisor-male subordinate (6.7%). Specifically, if the subordinate is a woman (*n* = 16,026), in most cases her supervisor is also a woman (53.2%), while if the subordinate is a man (*n* = 13,807), only a minority will have a woman as a supervisor (14.5%).

As for the number of workers in the respondent’s local workplace, the majority of employees - 53.4% - work in places with fewer than 50 employees. Specifically, 32.5% of employees work in a center with 10 to 49 workers - the rest are evenly distributed among the different intervals (from 2–4, 5–9, 50–99, 100–249, 250–499 to more than 500 workers), with the only exception being “single-worker centers” that account for only 2.2% of cases.

### 3.2 Measurement of variables

Dependent variable: subordinate job satisfaction. This paper uses a global measurement in which subordinates respond to the following question: “On the whole, are you very satisfied, satisfied, not very satisfied or not at all satisfied with working conditions in your main paid job?” The values of the 4-point answering format were recoded so that 1 corresponded to “not at all satisfied” and 4 to “very satisfied”. Single-item scales for measuring job satisfaction have been used in numerous papers in the past (Grissom et al., 2012; Campione, 2014) and have proven to be appropriate (Wanous et al., 1997).

Independent and moderating variables: supervisor supportive behaviors, supervisor gender, and supervisor-subordinate gender similarity. First, respondents specified their level of agreement, on a 5-point Likert-type answering format (recoded so that 5 corresponds to maximum agreement), with six statements about their immediate boss’s behavior: (a) “respects you as a person” (*Respect*); (b) “gives you praise and recognition when you do a good job’ (*Giving recognition*); (c) “is successful in getting people to work together” (*Coordinating work*); (d) “is helpful in getting the job done” (*Helping get the job done*); (e) “provides useful feedback on your work” (*Providing useful feedback*); (f) “encourages and supports your development” (*Encouraging development*). Second, referring to supervisor gender, respondents answered the following question: “Is your immediate boss a man or a woman?” We created a dummy variable, coding 0 = “man”; 1 = “woman”. Finally, we created a dummy variable for supervisor-subordinate gender similarity, coding 0 = different gender; 1 = same gender.

Control variables. We control for the effect of other variables that can have an impact on job satisfaction. Related to the subordinates: *age* (“How old are you?”, in years), *level of education* [(1) Early childhood education, (2) primary education, (3) lower secondary education, (4) upper secondary education, (5) post-secondary non-tertiary education, (6) short-cycle tertiary education, (7) bachelor or equivalent, (8) master or equivalent, (9) doctorate or equivalent], *seniority in the organization* (“How many years have you been in your company or organization?”, in years). Concerning employee occupation: *technicians and professionals* (dummy: 0 = no; 1 = yes) and *service and sales workers* (dummy: 0 = no; 1 = yes). Finally, referring the industry where employee’s organization operates: *private sector* (dummy: 0 = no; 1 = yes); and *manufacturing industry* (dummy: 0 = no; 1 = yes).

Previous studies have found that job satisfaction follows a U-shaped pattern with respect to an employee’s age (Clark et al., 1996). While new entrants to the labor market harbor positive feelings about their transition to adulthood, the growing boredom, and the sense of decreased opportunities during the first years of work lead them to lower job satisfaction; however, more mature workers tend to have more “attractive” jobs and greater power and status at work than younger employees, and so higher job satisfaction (Clark et al., 1996). Concerning the level of education, a positive effect on job satisfaction can be expected, since higher levels of education often lead to more attractive and rewarding jobs (Ng et al., 2005). Finally, it can be expected that people will become more satisfied as their seniority within a given organization increases, since it may result in more opportunities to gain promotion, status and power (Kalleberg and Mastekaasa, 2001). In addition, previous studies have also shown that workers in high-prestige occupations (e.g., technicians, professionals) have higher levels of job satisfaction (Hodson, 1989). With respect to industry, we expect that people working in the private sector will be less satisfied than those working for non-profit or public organizations as they mainly perform profit oriented work (Kumari and Pandey, 2011). Finally, and according to the evidence from Peccei and Lee (2005), people working in the manufacturing industry report lower job satisfaction than, for instance, those working in the health sector.

### 3.3 Data analysis

We first carried out a correlation analysis to study the interrelationships between the research variables and examine the possibility of multicollinearity. In order to contrast the hypotheses *H1*, *H2*, and *H3*, we run multiple linear regression analyses. We estimated one model with three steps, the dependent variable being *subordinate job satisfaction*. In the first step, a set of control variables were introduced: *Subordinate age*, *Subordinate level of education*, *Subordinate seniority in the company*, *Subordinate occupation-technicians and professionals*, *Subordinate occupation-service and sales*, *Firm industry-private sector*, and *Firm industry-manufacturing*. Concerning the control variable *Subordinate age*, which is expected to have a U-shaped relationship with job satisfaction, we introduced the original variable centered and its squared value into the model so that together they would produce the curvature. In the second step, we introduced the six *supervisor supportive behaviors* as independent variables to test *H1*. Finally, in the third step, we introduced *supervisor gender* and *supervisor-subordinate gender similarity* as independent variables to test *H2* and *H3*. We used collinearity diagnostics, in particular the variance inflation factor (VIF) and the condition index, to assess the instability potential of the regression coefficient.

Next, we used Hayes’s (2022) method, applying the PROCESS v4.2 macro in SPSS (specifically, PROCESS Model 1) to test *H4* concerning the moderating effects. We introduced the same set of control variables and estimated the moderating effect of supervisor-subordinate gender similarity on the relationship between each supervisor supportive behavior (independent variable) and subordinate job satisfaction (dependent variable). We estimated one Hayes model for each supervisor supportive behavior that has an impact on subordinate job satisfaction according to the linear regression analysis.

## 4 Results

Table 2 shows the correlations between the variables. The values show that there is no problem of multicollinearity since the highest and most statistically significant correlation is between the supportive behaviors of “Giving recognition” and “Encouraging development”, and has a score of 0.687, which is lower than the recommended limit of 0.75. The VI*F* value and condition index in the estimated regressions (Table 3) are below 10 and 30, respectively, which are the cut-off points recommended in the literature. These results suggest that there is no problem with multicollinearity in the data.

**Table 2:** Correlations, means, standard deviations, maximums and minimums (*n* = 29,833).

	1	2	3	4	5	6	7	8	9	10	11	12	13	14	15	16	17
1. Subordinate Job satisfaction	–																
2. Subordinate age (centered)	−0.013^*^	–															
3. Subordinate age (squared)	0.059^***^	0.055^***^	**–**														
4. Subordinate level of education	0.103^***^	−0.071^***^	−0.076^***^	–													
5. Subordinate seniority in the company	0.015^**^	0.535^***^	0.015^**^	0.020^***^	–												
6. Subordinate occupation: technicians and professionals (0 = no; 1 = yes)	0.121^***^	0.026^***^	−0.053^***^	0.543^***^	0.136^***^	–											
7. Subordinate occupation: service and sales (0 = no; 1 = yes)	−0.016^**^	−0.102^***^	0.071^***^	−0.158^***^	−0.137^***^	−0.328^***^	–										
8. Firm industry: private sector (0 = no; 1 = yes)	−0.059^***^	−0.154^***^	0.024^***^	−0.256^***^	−0.249^***^	−0.309^***^	0.082^***^	–									
9. Firm industry: manufacturing (0 = no; 1 = yes)	−0.053^***^	0.011	−0.038^***^	−0.126^***^	0.034^***^	−0.125^***^	−0.170^***^	0.247^***^	–								
10. Supervisor - Respect (a)	0.379^***^	0.003	0.042^***^	0.063^***^	0.019^***^	0.084^***^	−0.005	−0.049^***^	−0.060^***^	–							
11. Supervisor - Giving recognition (b)	0.378^***^	−0.036^***^	0.055^***^	0.068^***^	−0.023^***^	0.079^***^	0.016^**^	−0.025^***^	−0.067^***^	0.551^***^	–						
12. Supervisor - Coordinating work (c)	0.351^***^	−0.034^***^	0.046^***^	−0.010	−0.028^***^	0.010	0.019^**^	0.003	−0.037^***^	0.566^***^	0.578^***^	–					
13. Supervisor - Helping get the job done (d)	0.306^***^	−0.081^***^	0.014^*^	0.041^***^	−0.034^***^	0.050^***^	0.015^*^	0.017^**^	−0.048^***^	0.463^***^	0.527^***^	0.568^***^	–				
14. Supervisor - Providing useful feedback (e)	0.334^***^	−0.058^***^	0.038^***^	0.035^***^	−0.032^***^	0.042^***^	0.019^***^	0.003	−0.045^***^	0.523^***^	0.636^***^	0.610^***^	0.588^***^	–			
15. Supervisor - Encouraging development (f)	0.392^***^	−0.070^***^	0.034^***^	0.078^***^	−0.022^***^	0.101^***^	0.001	−0.037^***^	−0.052^***^	0.574^***^	0.687^***^	0.632^***^	0.603^***^	0.672^***^	–		
16. Supervisor gender (0 = man; 1 = woman)	0.020^***^	0.030^***^	0.013^*^	0.084^***^	−0.004	0.115^***^	0.128^***^	−0.178^***^	−0.128^***^	0.023^***^	0.059^***^	0.015^*^	0.016^**^	0.037^***^	0.052^***^	–	
17. Supervisor-subordinate gender similarity (0 = different gender; 1 = same gender)	−0.019^***^	0.013^*^	0.007	−0.089^***^	0.007	−0.059^***^	−0.012^*^	0.015^*^	0.024^***^	−0.014^*^	0.003	0.014^*^	0.020^***^	0.019^**^	0.011	0.203^***^	–
N	29,723	29,735	29,735	29,743	29,405	29,645	29,645	29,662	29,454	29,509	29,436	28,756	29,127	29,262	29,102	29,833	29,833
Minimum	1	−26.84	0.03	1.00	0.00	0.00	0.00	0.00	0.00	1.00	1.00	1.00	1.00	1.00	1.00	0.00	0.00
Maximum	4	0.46.16	2,130.75	9.00	64.00	1.00	1.00	1.00	1.00	5.00	5.00	5.00	5.00	5.00	5.00	1.00	1.00
Mean	3.04	−0.003	152.257	4.81	9.17	0.298	0.202	0.66	0.1475	4.40	3.85	3.95	3.70	3.85	3.83	0.35	0.681
Standard deviation	0.692	12.339	168.354	1.673	9.475	0.457	0.401	0.474	0.355	0.862	1.164	1.072	1.257	1.142	1.166	0.478	0.466

^*^p < 0.05, ^**^p < 0.01, ^***^p < 0.001.

**Table 3:** Estimated linear regression model of subordinate job satisfaction: direct effects.

Variables	Step 1: Control variables	Step 2: Control + supportive behavior effect	Step 3: Control + supportive behavior effect + supervisor gender and supervisor-subordinate gender similarity
Subordinate age (centered)	−0.021	−0.002	−0.001
Subordinate age (squared)	0.057^***^	0.033^***^	0.033^***^
Subordinate level of education	0.051^***^	0.042^***^	0.041^***^
Subordinate seniority in the company	0.009	0.009	0.008
Subordinate occupation: technicians and professionals (0 = no; 1 = yes)	0.092^***^	0.056^***^	0.057^***^
Subordinate occupation: service and sales (0 = no; 1 = yes)	0.011	0.002	0.004
Firm industry: private sector (0 = no; 1 = yes)	−0.011	−0.011	−0.013^*^
Firm industry: manufacturing (0 = no; 1 = yes)	−0.029^***^	−0.009	−0.010
Supervisor – Respect (a)		0.164^***^	0.164^***^
Supervisor – Giving recognition (b)		0.115^***^	0.116^***^
Supervisor – Coordinating work (c)		0.085^***^	0.085^***^
Supervisor – Helping get the job done (d)		0.033^***^	0.033^***^
Supervisor – Providing useful feedback (e)		0.011	0.011
Supervisor – Encouraging development (f)		0.130^***^	0.130^***^
Supervisor gender (0 = man; 1 = woman)			−0.012^*^
Supervisor-subordinate gender similarity (0 = different gender; 1 = same gender)			−0.012^*^
Δ*R*^2^	2.2%	19.4%	0.0%
Δ*F*	74.368^***^	1,099.700^***^	5.683^***^
*n*		26,694	
*R*^2^ final adjustment		21.6%	
*F*		459.629^***^	
Durbin-Watson		1.879	
VIF (min/max)		1.011/2.707	
Condition index		28.439	

^*^p < 0.05, ^**^p < 0.01, ^***^p < 0.001.

**Figure 2:**
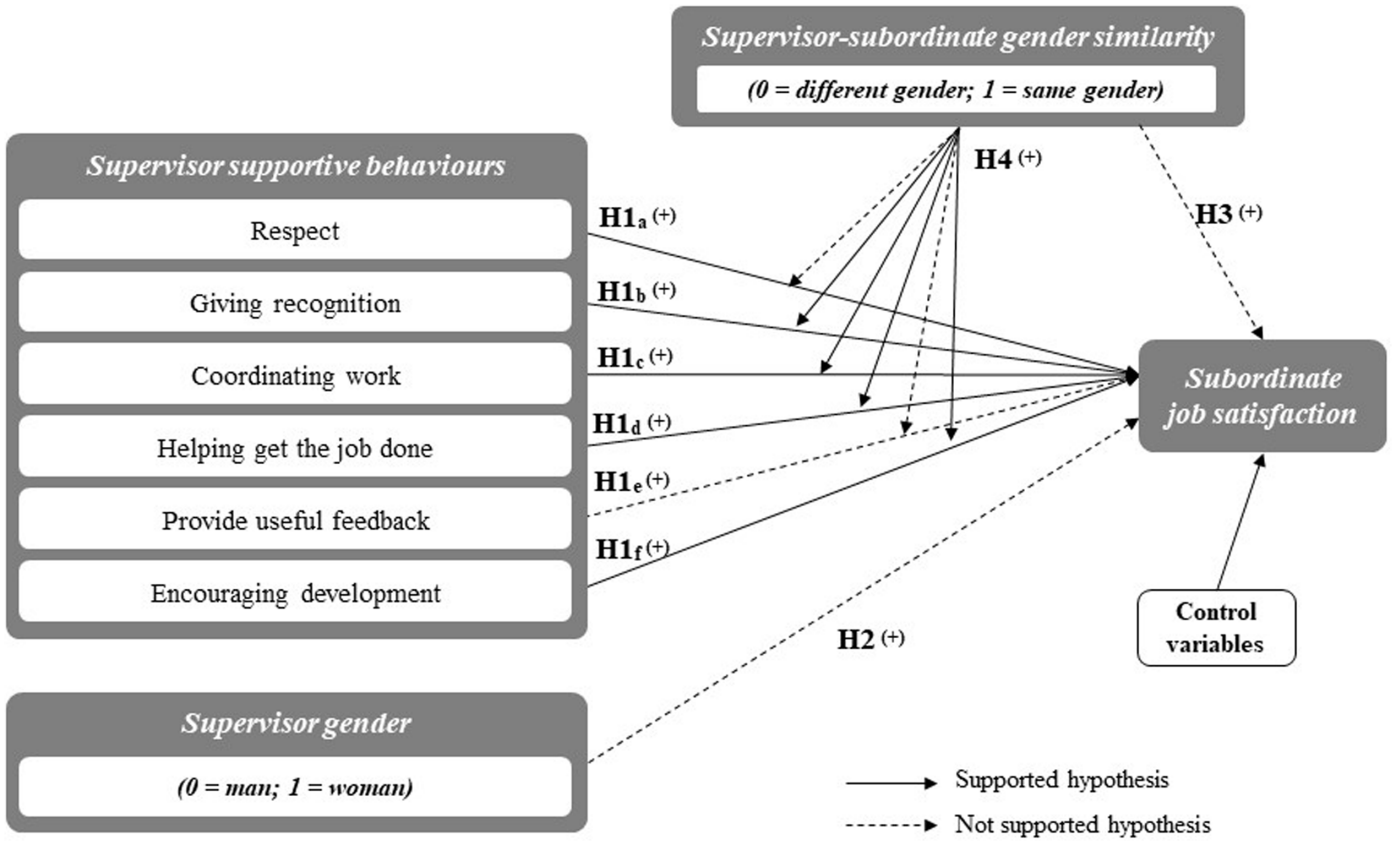
Graphical overview of the effects found.

Concerning hypotheses testing, Figure 2 summarizes the direct and moderating effects that found and did not find support. Table 3 shows the estimated regressions for analyzing the direct effect of supervisor supportive behaviors - variables *a* to *f* - (*H1*), as well as the direct effects of supervisor gender (*H2*) and supervisor-subordinate gender similarity (*H3*) on subordinate job satisfaction. Concerning the control variables, estimations in step 3 provide support to three of the expected effects of controls, with *subordinate age-squared* (*β* = 0.033, *p* < 0.001), *level of education* (*β* = 0.041, *p* < 0.001) and *occupation-technicians and professionals* (*β* = 0.057, *p* < 0.001) increasing job satisfaction. However, *seniority* and *occupation-service and sales* have a non-significant impact on job satisfaction (*β* = 0.008, *p* = 0.235; and *β* = 0.004, *p* = 0.532, respectively). Controls related to the industry in which the firm operates have a negative impact (i.e., *private sector*, *β* = −0.013, *p* = 0.038) or have a non-significant effect on job satisfaction (*manufacturing*, *β* = −0.010, *p* = 0.097).

In relation to *H1*, results (Table 3, step 3) show that all supervisor supportive behaviors have a positive and significant effect on subordinate job satisfaction, except for “Providing useful feedback”, which has a non-significant, standardized beta coefficient (*β* = 0.011, *p* = 0.167). Therefore, these results support *H1*a, *H1*b, *H1*c, *H1*d, and *H1*f, but not *H1*e (Figure 2).

Table 3 (step 3), further, shows a negative and significant impact of *supervisor gender* on *subordinate job satisfaction* (*β* = −0.012, *p* = 0.046). In other words, having a female supervisor reduces the job satisfaction of the subordinate. As this result is the opposite of the expected, *H2* does not find support (Figure 2).

In addition, Table 3 (step 3) also shows the direct effect of *supervisor-subordinate gender similarity* on *subordinate job satisfaction* (*H3*). The standardized beta coefficient reveals a negative and significant impact (*β* = −0.012, *p* = 0.027), which implies that having a supervisor of the same gender reduces the subordinate job satisfaction. Again, as this result is the opposite of the expected, *H3* does not find support (Figure 2).

Finally, Table 4 reports results of estimations using Hayes Model 1 for assessing the moderating effect of gender similarity on the relationship between the different supportive behaviors that have a significant impact on subordinate job satisfaction (i.e., the estimation was not run for providing useful feedback). Thus, we estimated five models and we found significant and negative moderating effects of gender similarity for the impact of the following supportive behaviors: *giving recognition* (*β* = −0.011, *p* = 0.099), *coordinating work* (*β* = −0.024, *p* = 0.002), *helping get the job done* (*β* = −0.012, *p* = 0.077) and *encouraging development* (*β* = −0.016, *p* = 0.023). These results indicate that the impact of supportive behaviors on job satisfaction is weakened in cases where gender similarity exists (Figure 2). Accordingly, *H4* finds support (Figure 2).

**Table 4:** The moderating effect of gender similarity on the impact of supportive behaviors on subordinate job satisfaction: estimations using Hayes Model 1.

Variables	Dependent variable: subordinate job satisfaction				
	Model a. Respect	Model b. Giving recognition	Model c. Coordinating work	Model d. Helping get the job done	Model f. Encouraging development
Subordinate age (centered)	−0.0013^**^	−0.0006^†^	−0.0007^†^	0.0002	0.0005
Subordinate age (squared)	0.0002^***^	0.0002^***^	0.0002^***^	0.0002^***^	0.0002^***^
Subordinate level of education	0.0163^***^	0.0151^***^	0.0236^***^	0.0187^***^	0.0161^***^
Subordinate seniority in the company	0.0005	0.0012^*^	0.0011^*^	0.0005	0.0006
Subordinate occupation: technicians and professionals (0 = no; 1 = yes)	0.1014^***^	0.0980^***^	0.1188^***^	0.1124^***^	0.0863^***^
Subordinate occupation: service and sales (0 = no; 1 = yes)	0.0101	0.0001	0.0083	0.0100	0.0038
Firm industry: private sector (0 = no; 1 = yes)	−0.0120	−0.0199^*^	−0.0224^*^	−0.0341^***^	−0.0127
Firm industry: manufacturing (0 = no; 1 = yes)	−0.0279^*^	−0.0241^*^	−0.0381^***^	−0.0307^**^	0.0351^**^
Supervisor-subordinate gender similarity (0 = different gender; 1 = same gender)	−0.0115	−0.0199^*^	−0.0234^**^	−0.0252^**^	−0.0236^**^
Supervisor – Respect (a)	0.3053^***^	–	–	–	–
Interaction 1.a (Respect^*^Similarity)	−0.0129	–	–	–	–
Supervisor – Giving recognition (b)	–	0.2247^***^	–	–	–
Interaction 1.b (Giving recognition^*^Similarity)	–	−0.0113^†^	–	–	–
Supervisor – Coordinating work (c)	–	–	0.2406^***^	–	–
Interaction 1.c (Coordinating work*Similarity)	–	–	−0.0237^**^	–	–
Supervisor – Helping get the job done (d)	–	–	–	0.1718^***^	–
Interaction 1.d (Helping get the job done*Similarity)	–	–	–	−0.0116^†^	–
Supervisor – Encouraging development (f)	–	–	–	–	0.2362^***^
Interaction 1.f (Encouraging development*Similarity)	–	–	–	–	−0.0156^*^
*n*	28,279	28,204	27,572	27,917	27,903
*R*^2^ final adjustment	15.73%	15.48%	14,45%	33.31%	16.57%
*F* value	479.69^***^	469.45^***^	423.21^***^	316.60^***^	503.77
Test of unconditional interaction (Δ*R*^2^)	0.01%	0.01%	0.03%	0.01%	0.02%
Test of unconditional interaction (Δ*F*)	1.92	2.72^†^	9.86^**^	3.12^†^	5.21^*^

†*p* < 0.10, ^*^p < 0.05, ^**^p < 0.01, ^***^p < 0.001. The estimation was not run for “providing useful feedback” because this supportive behavior showed no significant effect on job satisfaction according to the linear regression model (Table 3).

## 5 Discussion and conclusions

This study adds to the research on leadership by providing evidence from 29,833 subordinates from 35 European countries of the influence that six different supervisor supportive behaviors have on subordinate job satisfaction, while considering the gender of the individuals involved - the supervisor and the subordinate. In general, subordinates’ job satisfaction depends on their perceptions of the supportive behaviors provided by their supervisors in terms of respect, giving recognition for a job well done, coordinating work, helping get the job done, and encouraging their professional development. However, the effectiveness of those supportive behaviors-except for “respect”, which is the behavior that has the strongest impact on job satisfaction-depends on supervisor-subordinate gender similarity. Moreover, subordinates with female supervisors will experience lower job satisfaction, as well as those with a supervisor of the same gender.

Without considering the gender similarity between supervisor and subordinate (i.e., whether they are of the same gender or not), our paper provides evidence that five out of the six supervisor supportive behaviors considered in the present study increase subordinate job satisfaction: respect towards subordinates, recognition of a job well done, coordinating work, assistance in performing the job, and motivation for the development of their subordinates. Therefore, our results generally confirm the results of previous literature that suggests that supervisor supportive behaviors increase subordinate job satisfaction (Gilbreath and Benson, 2004; Qureshi and Hamid, 2017; Kizuki and Fujiwara, 2020; Hauff et al., 2022). However, the supportive behavior related to providing useful feedback seems to have no effect on job satisfaction. It is possible that feedback provided by the supervisor, especially in cases where it does not fit with the employee’s expectations (e.g., some criticism, even of the constructive variety), may be considered by the subordinate as a source of pressure and stress (e.g., to get the job done well), and so harms their job satisfaction. In this case, and according to the job demands-resources model, the feedback could be perceived more as a challenge than as support-in other words, it could be more of a job demand than a job resource (Bakker et al., 2007; Bakker and Demerouti, 2007). Therefore, a non-significant relationship can emerge due to the combination of positive and negative subjective experiences in receiving and facing such feedback. In this respect, London and Smither (2002) warn that an organizational culture which is supportive of feedback processes must be developed in order to get feedback to improve satisfaction.

Considering the gender of the supervisor as a visible attribute, and contrary to our hypothesis, this paper finds that the gender of the supervisor has a direct and negative effect on the subordinate’s job satisfaction, meaning that having a female supervisor reduces job satisfaction. This effect is found after controlling for variables like firm industry, subordinate occupation, and personal attributes such as age and level of education, and remains true regardless of subordinates’ perception of the supportive behavior received. Thus, the results contradict our assumptions based on social role theory (Eagly et al., 1995; Eagly and Wood, 2013) because the higher level of communal traits that female supervisors are supposed to have according to that theory, seems not to result in an increase in subordinate job satisfaction. This result could be explained by role congruence theory (Eagly and Karau, 2002), since what could be occurring is the stereotypic fit hypothesis. This hypothesis suggests that group members will experience discrimination in different social roles or positions to the extent that their group stereotypically does not have characteristics associated with success in those positions - e.g., stereotypically, women are often considered less competent and fitting for leadership positions than men. In the case of our research, it could be that subordinates are more satisfied in their job with a male boss, who stereotypically fits the desirable characteristics for that position (aggressive, ambitious, etc.).

The present study also finds an impact of *supervisor-subordinate gender similarity* on subordinate job satisfaction that was contradictory to our hypothesis, as when similarity exists, satisfaction decreases rather than increases. This result contravenes the similarity attraction framework (Schieman and McMullen, 2008), which suggests that “gender similarity” increases attraction and convergence of attitudes between supervisors and their subordinates, fostering cohesion and reducing potential conflicts at work. It also contradicts the idea that subordinates’ job satisfaction could be affected by the shared preferences for a particular style in the leader-member exchange - task-oriented style for men and people-oriented style for women (Fritz and van Knippenberg, 2020). Following Li et al. (2023), the results could be suggesting that in cases where the supervisor and subordinate are of the same gender, the former needs to maintain their status by remarking on the differences with their subordinates, which enlarges supervisors’ psychological distance with their subordinates of the same gender. This would explain why the subordinate does not feel the cohesion, convergence, and attraction that the similarity attraction framework suggests.

**Figure 3:**
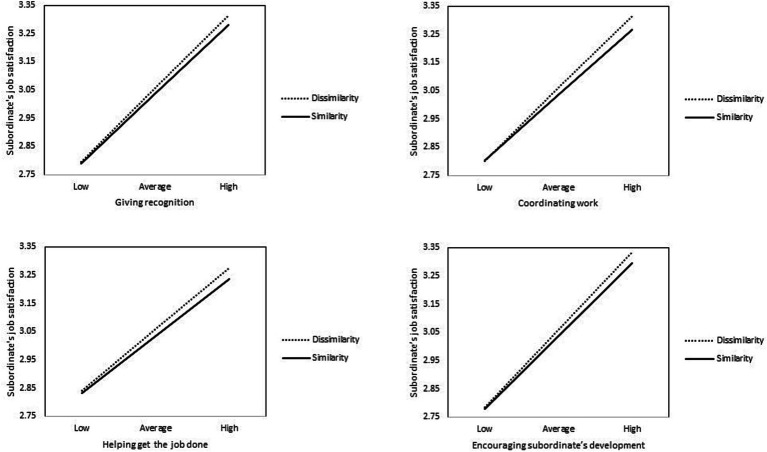
Moderating effect of gender similarity on the impact of supportive behaviors on subordinate’s job satisfaction.

Moreover, we found that four supervisor supportive behaviors that influence job satisfaction (i.e., giving recognition, coordinating work, helping get the job done and encouraging development) are moderated by supervisor-subordinate gender similarity. As showed in Figure 3, the moderation always has the same direction - that is, when similarity exists, the effect of those supportive behaviors on job satisfaction weakens, which is coherent with previous literature that has studied the moderating effect of gender similarity (Li et al., 2021, 2023). However, this moderating effect was not found for “respect” as a supportive behavior. It deserves to be highlighted that, according to previous literature, this supportive behavior is one of the most valued aspects of the subordinate’s daily experience at work (Decker and van Quaquebeke, 2015) and is often prioritized over other working conditions (van Quaquebeke et al., 2009). Indeed, in our sample, “respect” as perceived by employees, is the supervisor behavior that has more impact on subordinates’ satisfaction (see Table 3). These results suggest that “respect” can be considered a key and relevant supportive behavior for a trusting work environment that transcends the gender issues of the supervisor-subordinate relationship. Thus, our results are not only coherent with, but go beyond previous authors that underscore the relevance of this supportive behavior (van Quaquebeke et al., 2009; Decker and van Quaquebeke, 2015).

### 5.1 Theoretical implications

Based on social role theory, social exchange theory, the job demands-resources model, and the similarity attraction framework, this work provides a new approach to the study of the influence of supervisor supportive behaviors on subordinate job satisfaction by addressing the various types of supportive behavior separately, while considering the gender of individuals involved (supervisor and subordinate). In particular, our models address relationships that have been rarely considered by previous literature: (1) the influence of different supervisor supportive behaviors - considered separately, not in aggregate form - on subordinate job satisfaction; (2) the influence of supervisor gender, as observable trait, on subordinate job satisfaction; (3) the direct impact of supervisor-subordinate gender similarity on subordinate job satisfaction; and (4) the moderating effect of supervisor-subordinate gender similarity on the impact of supervisor supportive behaviors on subordinate job satisfaction.

First, contrary to expectations, not all supportive behaviors improve job satisfaction, and not all behaviors have the same effect on subordinate job satisfaction. On the one hand, providing feedback to subordinates may be perceived by them as a source of pressure and stress rather than as supervisor support, suggesting the use of the job demands-resources model in future studies to confirm this conjecture. On the other hand, giving respect at work has been identified as the most impactful supportive behavior in supervisor-subordinate relationships for enhancing job satisfaction, underscoring its crucial role in fostering a trusting work climate that transcends gender issues. Therefore, the differences found in the direct impacts of supervisors’ behaviors on subordinates’ job satisfaction confirm the relevance of analyzing supportive behaviors separately, going beyond the consideration of supervisor support as a unified construct (Ariza-Montes et al., 2019; Artz et al., 2020; Kizuki and Fujiwara, 2020; Hauff et al., 2022). Future research should consider this finding and study the impact of the different supervisor supportive behaviors towards subordinates on other outcome variables (e.g., motivation, organizational identification, commitment, engagement).

Second, another relevant theoretical contribution of this work is the finding that female supervisors in comparison to their male counterparts, despite being more communal according to social role theory, and so presumably more able to deploy supportive behaviors, do not achieve higher job satisfaction for their subordinates. This finding contradicts our initial hypothesis and could be explained by the different working conditions that women and men face. For instance, job satisfaction may be lower when the supervisor is female because working conditions may be worse in occupations where the supervisor is a woman (e.g., lower wages). Accordingly, future research should consider the potential impact of working conditions on job satisfaction to a greater extent in order to clarify the reason why having a female supervisor relates to lower job satisfaction.

Third, gender similarity matters, but contrary to our theoretical expectations, it is having someone of the opposite gender as a supervisor that increases job satisfaction. This result also needs further research in order to identify the specific factors that generate a gap between what theoretical models lead one to predict and what is actually happening in the workplace concerning the interaction between supervisors and their subordinates under a gender perspective. For instance, looking for complementarity when the genders are different, but strengthening the hierarchy when the genders are the same, as suggested by Li et al. (2023).

And fourth, gender similarity weakens the positive effect of support on satisfaction, which reinforces the usefulness of social role theory in combination with the “meet and un-meet expectations framework” (Phillips and Loyd, 2006; Penning de Vries and Knies, 2023) in improving our understanding of the effects of supervisor support towards subordinates on the job satisfaction of the latter. Therefore, further research could benefit from the use of the expectancy disconfirmation theory (Ilgen, 1971) by incorporating new variables (e.g., subordinates’ expectations about their supervisor support, subordinate comparisons of actual support with their expectations) in the analysis to better understand this key relationship.

### 5.2 Practical implications

This paper is also useful for professionals with management responsibility having provided evidence of the specific supportive behaviors that supervisors can deploy which will increase subordinate job satisfaction (i.e., respect, giving recognition, coordinating work, helping get the job done, encouraging development). Supervisors who are able to develop most of the supportive behaviors identified in this study will be able to contribute more towards creating and consolidating a sense of job satisfaction among the employees under their direction.

Moreover, gender - as a visible trait of the supervisor - also turns out to be relevant in explaining subordinates’ job satisfaction. In particular, since subordinates under the supervision of a woman have lower job satisfaction, female supervisors and their companies should be aware that their efforts to establish working conditions that favor job satisfaction may be to some extent ineffective. From this perspective, it would be advisable for female supervisors to analyze other aspects of their own behavior regarding their relationship with male and female subordinates in order to identify factors that may be contributing to a reduction in job satisfaction (efforts made to make hierarchical relationships apparent, unmet expectations, etc.).

This paper could also be useful for institutions of higher education in subjects related to business administration and management. Specifically, they can find evidence in our results to help develop didactic content that promotes the development of the managerial capabilities necessary to exercise efficient, equitable, and integrative leadership in human resources management. For example, didactic content should include theoretical and practical learning activities related to the six supportive behaviors under study, also considering nuances concerning the way feedback should be provided. In particular, practical learning activities should include a wide range of guest speakers that allow the supervisors’ and subordinates’ perspectives in the cases of gender similarity and dissimilarity to be understood. Role playing of supervisor-subordinate interactions could also be included to provide a hands-on experience and later assess and learn from the experience with gender similarity and dissimilarity situations.

### 5.3 Limitations

Among the limitations of our study, it is worth noting that job satisfaction has been assessed at the individual level. However, since the supervisor is usually responsible for a heterogeneous group of employees, it may be of interest for future research to analyze the influence of team dynamics on the level of group satisfaction with the supervisor’s behavior (male or female) as a team leader.

Like other possible future research lines, based on the evidence provided, in addition to its replication in other geographical and socio-cultural contexts, it is proposed that a more in-depth analysis be made of other possible moderating variables between supervisor gender and subordinate job satisfaction, both internal to the company (e.g., organizational culture, primary structure, work teams, etc.) and external (institutional factors related to normative, regulatory or sociocultural environments).

## Data availability statement

Publicly available datasets were analyzed in this study. This data can be found here upon registration: https://www.eurofound.europa.eu/surveys/european-working-conditions-surveys/sixth-european-working-conditions-survey-2015.

## Ethics statement

Ethical review and approval was not required for the study on human participants in accordance with the local legislation and institutional requirements. Written informed consent from the patients/participants or patients/participants legal guardian/next of kin was not required to participate in this study in accordance with the national legislation and the institutional requirements.

## Author contributions

AG-C and SS-O contributed to conception and design of the study, organized the database and performed the statistical analysis, and wrote the first draft of the manuscript. AG-C, SS-O, FG-P, and MM-M wrote sections of the manuscript. All authors contributed to the article and approved the submitted version.
